# Injectable Mineral Supplementation During the Transition Period Reduces Uterine Disease and Hypocalcemia and Enhances Humoral Immunity in Holstein Dairy Cows

**DOI:** 10.3390/ani16060956

**Published:** 2026-03-19

**Authors:** Raquel Sousa Marques, Filipe Aguera Pinheiro, Clara Satsuki Mori, Susan Suárez-Retamozo, Marcos Busanello, Rodrigo de Almeida, Bruno Sivieri Lima, Luc Durel, Viviani Gomes

**Affiliations:** 1 Department of Internal Medicine, College of Veterinary Medicine and Animal Science, University of São Paulo, Ave. Prof. Orlando Marques de Paiva, 87, São Paulo 05508-270, SP, Brazil; raquel.sousa.marques@usp.br (R.S.M.); filipe.ag@usp.br (F.A.P.); clarasat@usp.br (C.S.M.); susan.suarez@usp.br (S.S.-R.); 2 Department of Animal Science, Luiz de Queiroz College of Agriculture (ESALQ), University of São Paulo (USP), Piracicaba 13418-900, SP, Brazil; marcosbusanello@hotmail.com; 3 Department of Animal Science, Federal University of Paraná, Rua dos Funcionários, 1540, Curitiba 80035-050, PR, Brazil; ralmeida@ufpr.br; 4 Virbac Brazil, Ave. Queiroz Filho, 1560, Vila Leopoldina, São Paulo 05317-000, SP, Brazil; bruno.lima@virbac.com.br (B.S.L.); luc.durel@virbac.com (L.D.)

**Keywords:** ketosis, hypocalcemia, metritis, metabolism, immunity

## Abstract

The transition period in dairy cows is marked by metabolic, oxidative, and immune challenges that increase susceptibility to periparturient diseases. Injectable mineral supplementation (IMS) has been proposed to support immunometabolic adaptation by enhancing antioxidant capacity and immune function, with consistent associations with improved health outcomes but variable effects on production. Therefore, this study evaluated the effects of repeated intramuscular multi-mineral supplementation during the transition period on health, metabolic stress, immune status, and productive performance in Holstein cows. Supplementation was associated with lower odds of subclinical hypocalcemia on day 4 postpartum in primiparous cows (*p* = 0.02) and overall for persistent subclinical hypocalcemia (*p* = 0.03). Multiparous cows (*p* = 0.04) and the overall population (*p* = 0.01) showed consistent effects on metritis following IMS. Supplemented cows had improved metabolic and uterine health indicators without affecting energy metabolism-related disorders. Although no differences were detected for major postpartum health disorders, its main benefits may involve immune competence, oxidative regulation, and physiological resilience rather than energy balance.

## Simple Summary

The period around calving is one of the most challenging stages in the life of a dairy cow. Supporting cows during this transition may improve their health and overall performance. This study evaluated whether repeated injections of essential minerals during the transition period could improve health, immune status, and productivity in Holstein cows. The supplementation was associated with improved indicators of uterine health and calcium status, although it had limited effects on classical energy metabolism markers (e.g., NEFA, BHB) or disorders such as mastitis or ketosis. These findings suggest that injectable mineral supplementation may be a useful management strategy to improve health during the transition period, potentially reducing disease risk and supporting animal welfare in dairy herds.

## 1. Introduction

The transition period in dairy cows, encompassing the three weeks before and after calving, represents a critical physiological window marked by profound metabolic, endocrine, and immunological adaptations. Reductions in dry matter intake, negative energy balance, and increased oxidative stress converge to impair immune competence and increase susceptibility to periparturient disorders, particularly uterine and mammary infections [1]. These challenges are closely interconnected, as oxidative stress and metabolic imbalance directly influence immune cell function and inflammatory regulation during early lactation.

Adequate mineral availability is essential to sustain immune responsiveness, antioxidant defenses, and metabolic homeostasis during the transition period. However, even when diets are formulated to meet requirements, dietary mineral supplementation may not ensure consistent intake or bioavailability due to reduced feed consumption around calving, gastrointestinal antagonists, and marked inter-individual variability among cows [2]. In this context, injectable mineral supplementation has been proposed as a strategy to bypass gastrointestinal limitations and provide a rapid and predictable supply of essential minerals during this critical phase. Experimental studies have demonstrated rapid increases in circulating mineral concentrations following parenteral administration, along with improvements in antioxidant capacity and indicators of hepatic metabolic adaptation, although direct assessments of hepatic mineral reserves remain limited to a small number of studies [3,4].

Across the literature, injectable mineral supplementation has been primarily associated with immunometabolic responses indicative of improved metabolic adaptation rather than with consistent changes in classical circulating energy metabolites such as non-esterified fatty acids or β-hydroxybutyrate, which are largely driven by the physiological stage of lactation [4]. Improvements in antioxidant enzyme activity and redox balance have been repeatedly reported, supporting the concept that mineral supplementation modulates the cow’s capacity to cope with metabolic and oxidative stress during the periparturient period [3].

Evidence further indicates that injectable mineral supplementation enhances immune function in transition dairy cows, with the most consistent effects observed in innate immune competence. Studies evaluating peripheral leukocyte function have reported improvements in polymorphonuclear leukocyte responsiveness and associated antioxidant enzyme activity, indicating tighter coordination between immune readiness and oxidative balance [5]. Parenteral micronutrient supplementation administered around calving has also been associated with improved systemic and mammary immune responses, including modulation of inflammatory pathways, enhanced antioxidant status, and improvements in humoral immune markers such as immunoglobulin concentrations [6]. In contrast, evidence regarding adaptive cellular and humoral immune responses remains more heterogeneous across studies, emphasizing the importance of aligning measured immune domains with clearly defined outcomes.

From a clinical perspective, injectable mineral supplementation has been consistently associated with a reduced incidence of periparturient diseases, including metritis, retained fetal membranes, mastitis, and stillbirth, across multiple field studies conducted during the transition period [5,7]. These health benefits have frequently been documented in studies that simultaneously evaluated productive and reproductive outcomes, supporting improved disease resistance and health resilience rather than direct stimulation of performance.

Regarding milk production, several large-scale trials in high-producing dairy herds have reported no significant effects of injectable mineral supplementation on milk yield or energy-corrected milk, despite concurrent improvements in immune function and reductions in disease incidence [5,7]. Positive effects on milk yield or milk components have been reported exclusively under specific conditions, including heat stress or marginal mineral status, indicating that productive responses are context-dependent rather than consistent across production systems [5,7]. Similarly, evidence regarding reproductive performance remains inconsistent, with several studies reporting no significant effects on fertility parameters following injectable mineral supplementation, and occasional improvements observed only under defined environmental or management conditions [5,7].

Despite the growing body of evidence supporting the immunological and health benefits of injectable mineral supplementation, important gaps remain. Few studies have simultaneously integrated immune, oxidative, metabolic, and clinical outcomes within the same experimental framework, limiting the ability to link mechanistic responses to clinically relevant endpoints. In addition, parity-specific responses and the extent to which immunometabolic improvements translate into measurable health and production outcomes under commercial conditions remain incompletely characterized.

Therefore, we hypothesized that repeated mineral supplementation administered during the transition period would modulate metabolic stress, immune competence, and health outcomes in dairy cows. The objective of this study was to evaluate the effects of repeated intramuscular administration of a multi-mineral solution containing phosphorus, magnesium, potassium, copper, and selenium on health outcomes, metabolic stress biomarkers, immune status, and productive performance in Holstein cows during the transition period.

## 2. Materials and Methods

### 2.1. Animals and Management

This experimental field trial was conducted according to the procedures approved by the Committee on Ethics in the Use of Animals of the Faculty of Veterinary Medicine and Animal Science at the University of São Paulo (USP) (protocol #3886141020).

The animals utilized in this study were from a commercial herd located in São Pedro, São Paulo State, Southeastern Brazil (47°54′50″ W, 22°32′55″ S). The cows were enrolled in the trial from February 2021 to May 2021, and the follow-up period continued until December 2021. The farm had 400 milking Holstein cows. The cows were housed either in a free-stall cross-ventilation barn with robotic milking or in a free-stall barn with a rotary milking parlor and milked twice a day. On average, each cow produced 37.5 kg of milk per day, resulting in a total average daily production of 15,000 kg of milk. Animal diets were formulated by the farm’s nutritionist following the recommendations of the National Academies of Sciences, Engineering, and Medicine (2021) [8]. The dietary compositions that met the prepartum and postpartum requirements are presented in Supplementary Table S1. The nutrient determinations are shown in Supplementary Tables S2 and S3.

Intramammary dry cow therapy was administered between 70 and 60 days before the expected calving date. Dry cows were housed in specific lots inside a free-stall barn with a cross-ventilation system. The animals were vaccinated against clostridiosis (Fortress 7, Zoetis, Lincoln, NE, USA), neonatal diarrhea (ScourGuard 4KC, Zoetis, Lincoln, NE, USA), reproductive diseases (CattleMaster Gold, Zoetis, Lincoln, NE, USA), and *E. coli*-causing mastitis (JVAC, Boehringer Ingelheim, St. Joseph, MO, USA). They were moved to a second free-stall barn with a ventilation tunnel approximately 30 days before the expected calving date, where they were maintained until calving. After calving, the cows were directed to a free-stall cross-ventilation barn with robotic milking or kept in a free stall with a rotary milking parlor, depending on mammary gland health, cow adaptation to the robot, and milk production, among other factors. At this point, they were vaccinated against keratoconjunctivitis (Biokeratogen, Biogénesis Bagó, Buenos Aires, Argentina) and mastitis (JVAC, Boehringer Ingelheim, St. Joseph, MO, USA).

### 2.2. Composition of Experimental Groups and Treatments

A total of 289 cows were initially enrolled in the study after application of the inclusion criteria. Animal losses occurred over the extended experimental period, which spanned from three weeks before calving to 90 days in milk, totaling 120 days of observation. Cows that did not receive the three scheduled doses of injectable mineral supplementation or that calved less than 14 days after the first injection were excluded from the analysis. At the end of the study, 246 cows remained and were included in the final dataset. Cows were allocated to either the injectable mineral supplementation group (IMS; *n* = 127) or the non-supplemented control group (NIMS; *n* = 119) and were balanced according to the number of calves. Animals were further stratified by parity into primiparous (NIMS, *n* = 47; IMS, *n* = 53) and multiparous cows (NIMS, *n* = 72; IMS, *n* = 74).

A power analysis was conducted using PROC POWER in SAS to evaluate the statistical robustness of the study. Considering the observed mean milk yields of 39 and 40 kg/d for the control and treated groups, respectively, a standard deviation of 2.5 kg/d, and the respective sample sizes (*n* = 119 and *n* = 127), the analysis yielded a statistical power of 87.8% at a significance level of 0.05.

Cows in the NIMS group received 10 mL of 0.9% NaCl by intramuscular injection at day −14 ± 7 (D-14), calving (D0), and 14 ± 7 (D14) days in milk (DIM), and cows of the IMS group were dosed thrice, similarly to the control group schedule, by intramuscular injection with a commercial multi-mineral supplement (Fosfosal, Virbac, São Paulo, Brazil) that provided, per 10 mL dose, 538 mg of phosphorus (from C_3_H_7_Na_2_O_6_P sodium glycerophosphate, and NaH_2_PO_4_ monosodium phosphate), 30 mg of magnesium (from MgCl_2_ magnesium chloride), 31.5 mg of potassium (from KCl potassium chloride), 15 mg of copper (from CuCl_2_ copper chloride), and 10.0 mg of selenium (from Na_2_SeO_4_ disodium selenate). The treatment regimen was adapted from previous studies [5,9,10].

### 2.3. Health Monitoring

Diseases were identified and registered based on the farm protocols. Cows that failed to release the placenta within 24 h post calving were considered to have retained the placenta. Daily uterine inspection was performed to detect metritis during the first 21 days after DIM. Between 30 and 40 days after calving, the cows were subjected to gynecological evaluation using a vaginal speculum. The veterinarians responsible for reproductive management classified cows that exhibited serosanguineous, purulent, or fetid secretions as positive for endometritis. An ultrasonographic evaluation (Mindray, Shenzhen, China) was performed by the same veterinarian to confirm the rectal palpation findings, which included the presence of liquid content in the uterus.

Ketosis was diagnosed on farm using a ketometer (KetoVet Brazil; TaiDoc Technology, Taiwan, China) on days 5 (D5) and 10 (D10) postpartum. Animals were classified as positive on D5 and D10, according to the following intervals: 0–1.1 mmol/L, no ketosis; 1.2–2.8 mmol/L, subclinical ketosis; ≥2.9 mmol/L clinical ketosis [11]. Blood for the diagnosis of hypocalcemia was collected from the animals by puncturing the coccygeal vein or artery in plain tubes without anticoagulant on calving day (D0) and the fourth day postpartum (D4) to determine the total calcium concentration. The cutoff point adopted to classify animals with subclinical hypocalcemia was <2 mmol/L [12]. Classification included transient hypocalcemic, hypocalcemic, persistently hypocalcemic and delayed hypocalcemic [13].

Postpartum subclinical mastitis screening was performed between the 6th and 7th day postpartum using the California Mastitis Test (CMT). Cows with a score of 2 (++) or 3 (+++) were considered positive for mastitis, according to farm criteria, and were subsequently sampled aseptically for bacteriological analysis and antimicrobial susceptibility testing. The diagnosis of subclinical mastitis was established based on the isolation of at least one pathogen by microbiological culture. In addition, the Mastitis Detection Index of the robotic milking system (VMS, DeLaval International AB, Tumba, Sweden) was used as an auxiliary mastitis indicator during herd monitoring. This index considered conductivity, milking interval, and the presence of blood at the quarter level. Cows with an index greater than 1.8 were separated for further evaluation following the milk examination procedures by CMT as previously described. Traditional milk cultures and the analysis of antimicrobial susceptibility and resistance tests were performed based on National Mastitis Council (NMC) (1999) [14] and the guidelines of the Clinical and Laboratory Standards Institute (2005) [15].

### 2.4. Milk Production, Milk Quality and Reproductive Performance

Data collection began during the prepartum period, from 30 to 20 days before the expected calving date, and lasted up to 90 DIM. Milk yield in the rotary milking parlor, metritis and endometritis occurrence, and reproductive performance were extracted from Dairy Plan (GEA Farm Technologies, Drummondville, QC, Canada) databases. Milk yield data from the milking robot were extracted using DeLaval VMSTM V300 software during the 1st week of lactation. The weekly average milk yield was recorded over 12 weeks, and individual somatic cell count (SCCs) was recorded monthly.

Reproductive indices, such as days between the calving date and the 1st artificial insemination and the type of semen used (sexed or conventional), were recorded to verify whether they impacted the conception rate, as well as the outcomes of pregnancy checks to estimate pregnancy rates between experimental groups.

### 2.5. Evaluation of Metabolic–Immune and Oxidative Status

Subsets of paired NIMS (*n* = 32) and IMS (*n* = 34) cows were bled seven times during the transition period: 3 weeks before the expected calving date (wk − 3), 2 weeks before the expected calving date (wk − 2), 1 week before the expected calving date (wk − 1), around calving day (0), 1 week postpartum (wk + 1), 2 weeks postpartum (wk + 2), and 3 weeks postpartum (wk + 3). Blood samples were collected from the coccygeal vein using the Vacutainer system (Becton Dickinson, Franklin Lakes, NJ, USA).

All analyses were conducted as single assays, except for the haptoglobin and Immunoglobulin G (IgG) assays, which were performed in duplicate.

Serum concentrations of total bilirubin, direct bilirubin, cholesterol, glucose (GLU), triglycerides, total protein, non-esterified fatty acids (NEFAs), beta-hydroxybutyric acid (BHB), albumin, and aspartate aminotransferase (AST) were determined in a Labmax 240 Premium automatic biochemical analyzer (Labtest Diagnostica, Minas Gerais, Brazil). References to commercial kits are shown in Supplementary Table S4 [16,17,18,19].

Samples for haptoglobin (Hp) and IgG determination were collected using tubes containing a clot activator (Vacutube, Labor Import, São Paulo, Brazil, manufactured in China) to obtain blood serum, which was then centrifuged at 1500× *g* for 10 min and stored in duplicate at −40 °C. IgG concentrations in serum samples were measured using an in-house sandwich ELISA, according to the procedures described by Gomes et al. 2023 [20]. The concentration of haptoglobin was determined based on its ability to bind to hemoglobin [21] using spectrophotometry.

Oxidative stress was assessed by collecting samples in heparin tubes (10 mL) immersed in crushed ice, while EDTA tubes (4 mL) (BD Vacutainer K2 Ethylenediaminetetraacetic acid—EDTA, 3.6 mg REF367841^®^; BD Diagnosis, Franklin Lakes, NJ, USA) were stored in a separate insulated box at 4 °C for transportation from the farm to the laboratory. Blood preparation started two to three hours after sample collection due to the distance between farm and the laboratory. Blood in heparin was centrifuged at 1900× *g* for 15 min in a refrigerated centrifuge at 4 °C. Plasma was removed and stored in black microtubes (HS4323K; Heathrow Scientific, Vernon Hills, IL, USA) at −80 °C for the analysis of Total antioxidant status (TAS) and thiobarbituric acid–reactive substances (TBARS). Cell fraction was washed by adding buffered saline solution (NaCl; Na_2_HPO_4_; NaH_2_PO_4_; Milli-Q H_2_O) and centrifuged at 1900× *g* for 15 min in a refrigerated centrifuge at 4 °C. The leukocyte layer and the buffered saline solution were removed. This process was repeated until the leukocyte layer could not be distinguished or was reduced so that it did not interfere with removal of the red blood cell (RBC) mass. RBCs stored in black microtubes were kept in the freezer at −80 °C for future determination of Glutathione peroxidase (GPx).

Blood in EDTA was added to tubes with distilled water and precipitant solution (HPO_3_; Na_2_EDTA·H_2_O; NaCl; H_2_O) after vortexing for homogenization for Reduced glutathione (GSH) analysis. The samples were rested for 5 min and then centrifuged at 1900× *g* for 5 min. After centrifugation, the intermediate layer was removed and stored in amber microtubes (Thermo Fisher Scientific, Dublin, Ireland) and stored at −80 °C.

Reduced glutathione levels were determined according to a previously described method [22]. Serum GPx activity (RS504 and RS505, Randox Brasil Ltd., São Paulo, Brazil) and TAS (NX2332, Randox Brasil Ltd., São Paulo, Brazil) determination were performed using commercial Randox test kits in an automated biochemical analyzer (Labmax 240 Premium, Labtest Diagnostica, Minas Gerais, Brazil). TBARS was assayed by dissolving 3% (*w*/*v*) 5-sulfosalicylic acid hydrate and thiobarbituric acid (TBA) solution at 0.67% in purified water at 95 °C for 30 min. The pH 1.8 was adjusted from 1.8 to 2.0 using a 1 M sodium hydroxide solution. For TBARS quantification, 0.25 mL of serum or washed erythrocytes were added to a test tube containing 0.25 mL of 3% 5-sulfosalicylic acid hydrate, and vortexed for 10 s, centrifuged at 18,000× *g* for 3 min, and left to rest for 15 min at 25 °C. Subsequently, 0.25 mL of purified water (blank) or supernatant (samples) was diluted into 0.5 mL of 0.67% TBA solution. The mixture was heated at 95 °C for 30 min and then cooled on ice for 10 min to stop further reaction. After blanks and samples were equilibrated to room temperature, 300 µL was pipetted into a microplate well and absorbance was measured at 535 nm. The results are expressed as nM TBARS per milligram of hemoglobin (Hb) or total protein (nM/mg of Hb or total protein) in washed erythrocytes or serum samples, respectively. The MDA-TBA complex extinction coefficient of 156,000 M^−1^/cm^−1^ at 25 °C and 0.9 cm path length was used for the calculations.

### 2.6. Statistical Analysis

A total of 289 animals were initially included in this study; however, 43 were removed for statistical analysis because they did not receive the complete 3-dose treatment and the interval between the first IMS treatment in the prepartum period and parturition was less than 14 days after the first injection. The final sample size is 246.

Analyses were performed using the Statistical Analysis System for Windows software (SAS version 9.4, SAS Institute Inc., Cary, NC, USA) and statistical significance was considered when *p* < 0.05 for all performed analyses. Descriptive statistics referring to qualitative nominal data (occurrence of diseases and pregnancy) were calculated using the SAS PROC FREQ. The occurrence of diseases was analyzed using a generalized linear mixed model with a binomial distribution and logit link function, fitted using the SAS PROC GLIMMIX. The model included treatment group as a fixed effect, with the “Control group” used as the reference category. This analysis was performed for general data and separately for primiparous and multiparous cows. Odds ratios were estimated to quantify the effect of treated group relative to the control. Least squares means were obtained on the probability scale using the inverse link function, and pairwise comparisons among groups were performed using differences in least squares means.

On the other hand, quantitative variables, like biochemical, metabolic, and milk yield, were evaluated using a general mixed model (SAS PROC GLIMMIX) considering fixed effects of group, sampling time (week or days in milk for milk yield), and their interaction. Repeated measurements within cows were accounted for by modeling the residual covariance structure at the cow level being that a random effect. Several variance-covariance matrixes were tested and those resulting in a lower corrected Akaike information criteria were used in the final modeling for each variable. Student’s *t*-test was used to verify statistical differences between groups.

All model assumptions were tested and satisfied (residual normality, homogeneity, and independence) using residual graphs (Q-Q plots, histograms, plots of residuals vs. predicted values). Somatic cell count was analyzed using the same model; however, due to its non-normal distribution, a generalized linear mixed model (SAS PROC GLIMMIX) assuming a lognormal distribution was used (dist = lognormal). Following the analysis, least squares means and their associated standard errors, originally estimated on the log scale, were back-transformed to the original scale. Re-transformed means were calculated as the exponentiated linear predictor adjusted by a bias correction factor exp(σ^2^/2) to obtain arithmetic means. The corresponding standard errors were derived from the back-transformed variance, accounting for the properties of the lognormal distribution.

## 3. Results

### 3.1. Descriptive Statistics

No significant association was observed between groups and calving difficulty (chi-square test, *p* > 0.05). The incidence of twin births, stillbirths, and the sex of the calves from the 246 experimental cows are shown in Table 1.

### 3.2. Health Monitoring

The incidence of postpartum diseases is shown in Table 2. The incidence of metritis, among multiparous cows, was lower in the IMS group compared with the NIMS group, representing a 55% lower odds of metritis (OR = 0.45, 95% CI = 0.21–0.95, *p* = 0.04). In the overall population, metritis was also less frequent in IMS cows than in NIMS cows, corresponding to a 58% reduction in odds (OR = 0.42, 95% CI = 0.23–0.78, *p* = 0.01). The incidence rates of retained placenta and endometritis were similar between the IMS and NIMS groups.

The incidence of ketosis on day 5 and day 10 postpartum did not differ between IMS and NIMS cows (*p* > 0.05). The odds of developing subclinical hypocalcemia on day 4, among primiparous cows, were 77% lower in the IMS group compared with the control group (OR = 0.23, 95% CI: 0.06–0.79). Additionally, in this group, treatment effects were observed for persistent subclinical hypocalcemia (*p* = 0.03), with IMS cows exhibiting 66% lower odds of the condition (OR = 0.34, 95% CI: 0.13–0.90).

No differences were observed between treatments for clinical mastitis, positive CMT, first AI status, and other diseases (*p* > 0.05).

### 3.3. Milk Production, Milk Quality and Reproductive Performance

**Figure 1:**
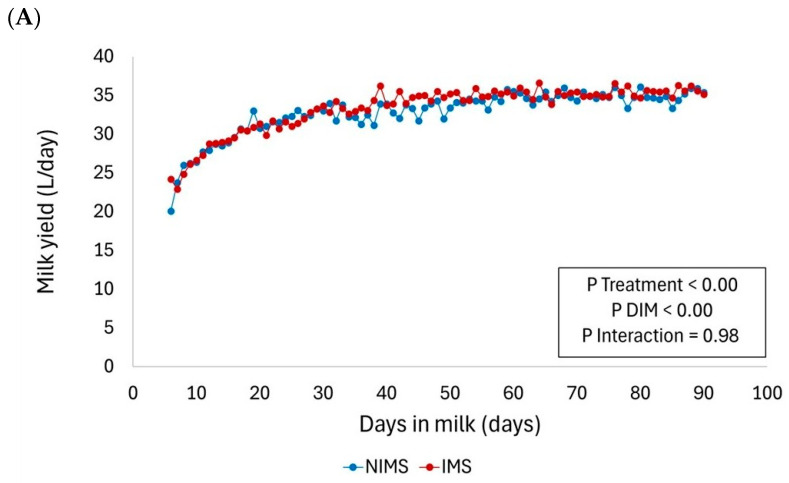

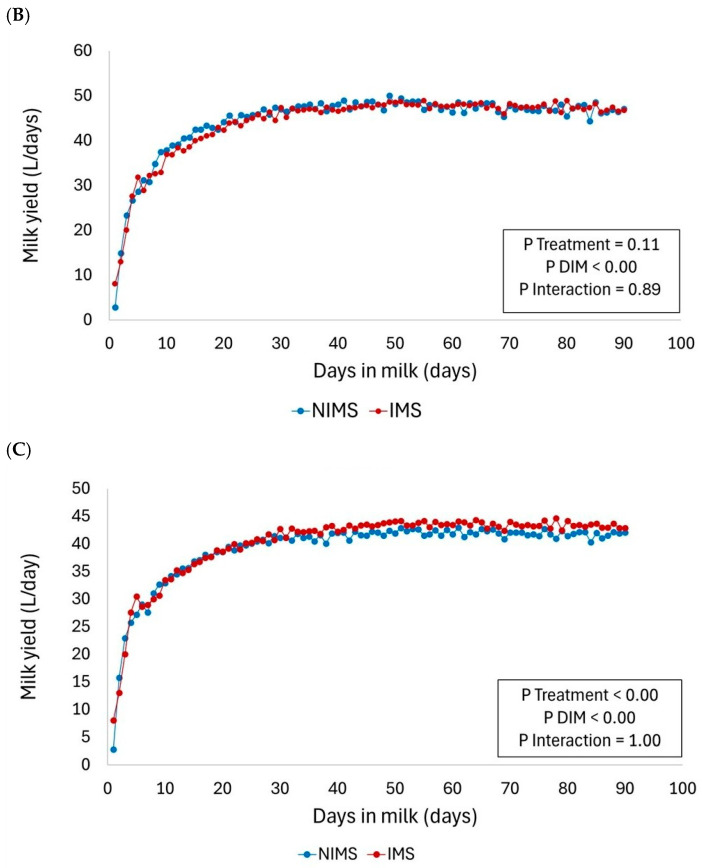
Milk production (L/d) over days in milk in cows from the NIMS and IMS groups within the primiparous category (**A**), the multiparous category (**B**) and the overall population (**C**). Abbreviations: NIMS = Non-supplemented control group; IMS = Injectable mineral supplementation group. Statistical analyses were performed using SAS PROC GLIMMIX with group, days in milk, and their interaction.

The effect of IMS treatment on milk production is shown in Figure 1A–C. In primiparous cows, across the evaluated period, IMS increased milk yield without altering the lactation pattern. Among multiparous cows, across the evaluated period, milk yield was numerically greater in the NIMS group (44.4 ± 0.17) than in the IMS group (44.0 ± 0.15); however, this difference was not statistically significant (*p* = 0.11). Days in milk significantly influenced milk production (*p* < 0.00), whereas no group × days in milk interaction was observed (*p* = 0.89), indicating that both groups followed a similar lactation pattern.

Milk yield increased rapidly during early lactation in both groups, reaching a plateau at approximately 30 to 40 days in milk. Throughout the evaluated period, cows in the IMS group consistently exhibited greater milk production (40.2 ± 0.16) than those in the NIMS group (39.2 ± 0.17). The group effect was significant (*p* < 0.00), and days in milk also significantly influenced milk yield (*p* < 0.00). The interaction between group and days in milk was not significant (*p* = 1.00), indicating that the treatment increased milk production without altering the lactation pattern. Both groups displayed a similar lactation curve, with stable milk production after peak yield. Day-to-day variation was minimal, and no abrupt declines in milk production were observed.

Differences in the incidence of SCC categories between treatments were observed. Overall, the group IMS showed greater SCCs at specific days in milk; however, SCC values were generally low (Table 3).

No differences were observed between treatments for reproductive indices such as days between the calving date and the 1st artificial insemination (*p* > 0.05).

### 3.4. Evaluation of Metabolic–Immune and Oxidative Status

No significant differences were observed between NIMS and IMS cows for the metabolic and inflammatory biomarkers evaluated relative to calving (Table 4).

In primiparous cows, no significant differences were observed between the NIMS and IMS groups for any of the evaluated metabolic or inflammatory biomarkers, as the group effect was not significant for all variables (Table 4). However, sampling time relative to calving significantly affected total protein (TP) (*p* = 0.00), globulin (GLOB) (*p* < 0.00), and triglycerides (TRIGL) (*p* = 0.02). No significant group × sampling time interactions were detected for any variable, suggesting similar temporal patterns between groups. In multiparous cows, no significant differences between NIMS and IMS groups were detected for the evaluated biomarkers (*p* > 0.05). In contrast, sampling time had a significant effect on several metabolic variables, including BHB, NEFA, TP, GLOB, AST, total bilirubin (TB), direct bilirubin (DB), TRIGL, and glucose (GLU) (*p* < 0.05) (Table 4).

When considering the overall population, no significant differences were observed between the NIMS and IMS groups for any of the evaluated metabolic or inflammatory biomarkers, as the group effect was not significant for all variables (*p* > 0.05). In contrast, sampling time relative to calving significantly affected all evaluated biomarkers except albumin (ALB) (*p* < 0.05) (Table 4).

No differences were observed between groups for haptoglobin (*p* > 0.05). Immunoglobulin G concentrations were affected by both sampling time and treatment. In primiparous cows, IgG varied according to sampling time (*p* < 0.00), and a treatment effect was identified, with differences between the NIMS and IMS groups (*p* = 0.02). Among multiparous cows, IgG concentrations also differed between treatments (*p* = 0.01) and according to sampling time (*p* < 0.00), with a significant treatment effect detected (*p* = 0.03). In the overall population, IgG showed a strong sampling effect (*p* < 0.00), and a significant treatment effect was observed, with differences between the NIMS and IMS groups (*p* = 0.00) (Table 5).

Using a 5% significance threshold, sampling time significantly influenced several oxidative stress-related biomarkers (Table 5). In primiparous cows, significant sampling effects were detected (*p* = 0.06), with total antioxidant status (TAS; *p* < 0.00) varying according to sampling time. Among multiparous cows, pronounced sampling effects were observed for thiobarbituric acid-reactive substances (TBARS; *p* < 0.00) and TAS (*p* < 0.00). In the overall population, consistent sampling effects were identified for glutathione peroxidase (GPX; *p* = 0.01), TBARS (*p* < 0.00), reduced glutathione (GSH; *p* = 0.01), and TAS (*p* < 0.00) (Table 5), indicating marked temporal variation in oxidative status during the transition period.

## 4. Discussion

The present study addressed whether repeated injectable multi-mineral supplementation during the transition period could improve the health outcomes, immunometabolic status, oxidative balance, and productive performance of Holstein cows managed under commercial conditions. However, direct comparison among studies evaluating injectable mineral supplementation remains challenging because of substantial heterogeneity in mineral formulations, trace element concentrations, inclusion of vitamins, dosing frequency, and timing relative to calving or other physiological stressors, all of which critically influence biological responses and may help explain the variability reported across the literature.

In this context, it is important to distinguish studies using formulations and protocols comparable to the present trial from those employing distinct mineral blends or intervention strategies. The injectable mineral formulation evaluated here provides phosphorus, selenium, copper, magnesium, potassium, and sodium and was administered repeatedly at −14 ± 7 d prepartum, calving, and 14 ± 7 DIM. This protocol was adapted from Enjalbert et al. (2006), Soldá et al. (2017), and Silva et al. (2022), who similarly targeted the transition period using repeated parenteral mineral administrations [5,9,10].

Studies by Soldá et al. (2017) and Silva et al. (2022) are therefore the most appropriate comparators, as they used multi-mineral injectable products during the transition period and evaluated health, metabolic, immune, and oxidative outcomes [5,10]. Other studies using injectable trace minerals enriched with zinc and manganese, or combined with vitamins A and E, provide mechanistic insights but must be interpreted with caution when extrapolating to the present formulation.

Injectable mineral supplementation was associated with reduced odds of metritis in the present study, particularly in multiparous cows. This finding agrees with reports by Soldá et al. (2017) and Silva et al. (2022), who observed improvements in postpartum health outcomes following injectable mineral supplementation during the transition period [5,10]. In contrast, some studies evaluating injectable trace minerals did not detect consistent reductions in uterine disease, particularly in herds with low baseline incidence or under optimal nutritional management. This divergence reinforces that baseline health and mineral adequacy strongly modulate responsiveness, rather than contradicting the biological plausibility of mineral-supported uterine defense.

Mechanistically, improved uterine health is consistent with the role of minerals such as selenium, copper, and zinc in supporting neutrophil migration, phagocytic capacity, oxidative burst regulation, and tissue-level immune responses. Although leukocyte functional assays were not evaluated in the present study, the reduction in metritis aligns with studies demonstrating enhanced innate immune function following injectable mineral supplementation [5].

The reduction in subclinical hypocalcemia in primiparous cows and the lower incidence of persistent hypocalcemia observed in the overall population are clinically relevant. While Fosfosal^®^ is not a calcium product, improved mineral status and metabolic support during the transition period may indirectly facilitate calcium homeostasis and postpartum adaptation. Persistent subclinical hypocalcemia has been linked to downstream disease risk and impaired productivity [13], suggesting that even modest reductions in its duration may contribute to improved transition resilience.

In contrast to health and calcium-related outcomes, injectable mineral supplementation had limited effects on NEFA, BHB, and ketosis incidence, except for a modest increase in glucose. These findings diverge from studies reporting improvements in metabolic biomarkers following injectable mineral supplementation, particularly under conditions of greater nutritional or environmental challenge [7,23]. In contrast, our results are consistent with reports showing minimal or inconsistent effects of injectable mineral supplementation on energy metabolism in well-managed herds, even when health or immune-related benefits were observed [5,10]. This divergence likely reflects that core drivers of negative energy balance, such as intake depression, adipose mobilization, and endocrine regulation, are not directly governed by micronutrient supply unless mineral deficiencies are limiting metabolic pathways.

A consistent finding of the present study was higher serum IgG concentrations in supplemented cows. Serum IgG concentrations were higher in supplemented cows across parities, indicating an effect of injectable mineral supplementation on humoral immune status. This result is highly concordant with multiple studies demonstrating that injectable mineral supplementation—particularly formulations containing selenium—enhances humoral immune responses. While immune responses to injectable mineral supplementation are not consistently reported across studies, several investigations have demonstrated immune modulation following parenteral micronutrient administration, particularly when selenium is included in the formulation [5,24].

Importantly, experimental studies evaluating injectable organic selenium sources provide strong biological evidence supporting the role of selenium in stimulating humoral immunity. Supplementation with diphenyl diselenide during the transition period has been shown to promote significant increases in serum total protein, globulins, and IgG concentrations in dairy cows, counteracting the physiological decline in immunoglobulins commonly observed postpartum [25]. Similar effects were reported in the offspring after the passive immune transfer reinforcing the immunomodulatory potential of this selenium source [26].

Although the selenium formulation and experimental design of these studies differ substantially from the commercial product used in the present trial, their findings highlight the sensitivity of humoral immune responses to parenteral selenium supply. Notably, increased IgG concentrations may have functional implications beyond humoral immunity alone. Immunoglobulins play a central role in opsonization and pathogen recognition, thereby enhancing innate immune mechanisms such as neutrophil activation and phagocytosis. Improved coordination between humoral and innate immune responses during early lactation may therefore represent a key pathway linking injectable mineral supplementation to reduced uterine disease incidence.

This interpretation is further supported by studies demonstrating improved innate immune function following injectable mineral supplementation, including enhanced polymorphonuclear leukocyte activity and antioxidant defenses [5]. Although functional immune assays were not performed in the present study, the increase in IgG concentrations and reduction in metritis incidence are biologically consistent with a scenario in which injectable mineral supplementation strengthens immune competence during the critical postpartum period.

Palomares et al. (2016) and Bittar et al. (2018) demonstrated that injectable trace minerals administered concurrently with vaccination resulted in earlier and more robust antigen-specific antibody responses, including greater neutralizing antibody titers against BVDV and BHV1 compared with non-supplemented controls [27,28]. These effects were observed following both parenteral and intranasal vaccination protocols, indicating a broad immunomodulatory role of injectable minerals.

Similarly, Mattioli et al. (2020) showed that parenteral supplementation with minerals and vitamins increased antibody titers following vaccination in calves, while maintaining antioxidant capacity [29]. Together, these studies indicate that injectable mineral supplementation enhances not only total IgG concentrations but also antigen-specific antibody production in response to immune challenge.

Beyond serving as a quantitative marker, IgG plays multiple functional roles: generation of neutralizing antibodies that block pathogen entry, opsonization to enhance phagocytosis, activation of the classical complement pathway, and facilitation of antibody-dependent cellular cytotoxicity. Increased IgG availability may therefore amplify cross-talk between humoral and innate immunity, potentially enhancing early pathogen clearance and contributing to reduced uterine disease risk.

Enhanced IgG availability may indirectly support innate immune function by improving opsonization efficiency and complement activation, thereby facilitating neutrophil-mediated bacterial clearance. This functional integration between humoral and innate immunity may represent a key mechanistic link between higher IgG concentrations and reduced metritis incidence observed in the present study.

The increase in GPx activity in multiparous cows aligns with the established role of selenium as a cofactor of glutathione peroxidase. Similar antioxidant responses have been reported by Silva et al. (2022) and Mattioli et al. (2020) [5,29], although not universally across studies. Parity-specific effects likely reflect the greater oxidative and inflammatory burden experienced by older cows during the transition period.

Unlike uterine health, no differences were observed in mastitis incidence or CMT positivity. This contrasts with findings by Soldá et al. (2017), who reported effects on SCCs [10]. However, the present herd exhibited low baseline SCCs and favorable udder health, limiting the ability to detect the effect of IMS on udder health. Under such conditions, lack of response does not negate potential benefits in herds with higher mastitis pressure. Regarding milk yield, our study agrees with Soldá et al. (2017) in terms of enhanced milk production in the IMS group [10].

Regarding milk yield, the present study showed a parity-dependent response, with greater milk production observed in primiparous cows receiving injectable mineral supplementation, whereas no significant differences were detected in multiparous cows. These findings partially align with the heterogeneous responses reported in the literature. Studies evaluating multi-mineral supplementation have reported a tendency toward greater milk yield in supplemented cows, suggesting that productive responses may occur under certain conditions [7]. However, these effects are not consistently detected across studies. For instance, Silva et al. (2022) reported no significant effect of injectable trace mineral supplementation on milk yield in dairy cows during the transition period, despite improvements in immunometabolic parameters [5].

In contrast, studies evaluating broader parenteral micronutrient strategies have reported productive responses under specific conditions. For example, Somagond et al. (2025) observed increased milk yield when cows received a combined parenteral supplementation of multivitamins and multi-minerals [24]. Taken together, these findings suggest that productive responses to injectable or parenteral micronutrient supplementation are influenced by factors such as nutrient formulation, physiological status, and herd management conditions. In this context, the lack of a significant milk yield response in multiparous cows in the present study should be interpreted as a context-dependent finding rather than as a contradiction of the biological plausibility of supplementation effects.

As discussed above, this study was conducted in a single, highly technified herd with excellent nutritional management, oral mineral supplementation, diets formulated according to NASEM recommendations, and low disease pressure. These conditions likely constrained the magnitude of observable responses, particularly for metabolic and inflammatory outcomes. However, a study limitation is that baseline mineral nutritional status was not measured; therefore, it was not possible to determine whether the observed responses reflected correction of pre-existing deficiencies or broader supplementation effects. In herds facing greater nutritional imbalances, higher pathogen exposure, or environmental stress, injectable mineral supplementation may yield more pronounced effects.

In summary, the present findings indicate that repeated injectable mineral supplementation during the transition period selectively improved uterine health, reduced calcium-related disorders, enhanced humoral immune status, and increased antioxidant enzyme activity, while exerting limited influence on classical energy metabolism markers. Comparative analysis across studies highlights that formulation, protocol, baseline mineral adequacy, and herd context critically shape responsiveness. Collectively, the evidence supports injectable mineral supplementation as a strategy to enhance transition resilience primarily through immune and oxidative pathways rather than direct modulation of energy metabolism.

## 5. Conclusions

The present study demonstrates that repeated injectable multi-mineral supplementation during the transition period confers targeted health and immune advantages in Holstein dairy cows. Injectable mineral supplementation had a positive effect on milk production in primiparous cows and reduced uterine disease risk, mitigated subclinical and persistent hypocalcemia, enhanced humoral immunity, and improved antioxidant enzyme activity, particularly in multiparous animals. These effects were not accompanied by consistent changes in classical indicators of energy metabolism or systemic inflammation, underscoring that the primary benefits of injectable mineral supplementation lie in improved immune competence, oxidative regulation, and physiological resilience rather than in direct modulation of energy balance.

## Figures and Tables

**Table 1:** Incidence of twins, stillbirths, and gender of the calves from Holstein dairy cows included in the study. No difference was found for the variables (*p* > 0.05). Statistical comparison between groups was made by using Chi-square test.

Product	Group		Total (%)
	NIMS (%)	IMS (%)	
Female	60.5	66.14	63.41
Male	32.78	28.35	30.49
Twins	3.36	3.94	3.66
Stillbirth	3.36	1.57	2.44

Abbreviations: NIMS = Non-supplemented control group, *n* = 119; IMS = Injectable mineral supplementation group, *n* = 127.

**Table 2:** Mean values and standard error (SE) for incidence of postpartum diseases in NIMS and IMS groups, expressed as mean ± SEM, along with corresponding odds ratios, 95% confidence limits, and *p*-value (5%).

Parity	Variables	Group (Mean ± SE)		Odds Ratio	(95% CI)	*p*-Value
		NIMS	IMS			
Primiparous	Retained placenta	0.11 ± 0.04	0.08 ± 0.04	0.69	0.17–2.77	0.59
	Metritis	0.26 ± 0.06	0.11 ± 0.04	0.37	0.13–1.10	0.07
	Endometritis	0.19 ± 0.05	0.19 ± 0.06	0.98	0.36–2.71	0.97
	Ketosis D5	0.13 ± 0.05	0.21 ± 0.06	1.79	0.60–5.36	0.30
	Ketosis D10	0.13 ± 0.05	0.17 ± 0.05	1.40	0.45–4.33	0.56
	Subclinical hypocalcemia D0	0.17 ± 0.06	0.14 ± 0.05	0.72	0.22–2.41	0.59
	Subclinical hypocalcemia D4	0.31 ± 0.07	0.09 ± 0.04	0.23	0.06–0.79	0.02
	Persistent subclinical hypocalcemia	0.05 ± 0.04	0.00 ± 0.00	-	-	0.97
	Subclinical mastitis	0.11 ± 0.04	0.09 ± 0.04	0.88	0.23–3.29	0.84
	Positive CMT	0.09 ± 0.04	0.23 ± 0.06	3.15	0.92–10.71	0.07
	First AI status	0.62 ± 0.07	0.56 ± 0.07	0.78	0.34–1.81	0.56
	Other diseases	0.02 ± 0.02	0.04 ± 0.03	1.80	0.15–21.19	0.64
Multiparous	Retained placenta	0.11 ± 0.04	0.05 ± 0.03	0.46	0.13–1.61	0.22
	Metritis	0.36 ± 0.06	0.20 ± 0.05	0.45	0.21–0.95	0.04
	Endometritis	0.15 ± 0.04	0.15 ± 0.04	1.06	0.42–2.72	0.90
	Ketosis D5	0.43 ± 0.06	0.31 ± 0.05	0.59	0.30–1.19	0.14
	Ketosis D10	0.43 ± 0.06	0.31 ± 0.06	0.61	0.30–1.21	0.16
	Subclinical hypocalcemia D0	0.50 ± 0.06	0.39 ± 0.06	0.64	0.32–1.29	0.21
	Subclinical hypocalcemia D4	0.28 ± 0.05	0.31 ± 0.06	1.17	0.55–2.50	0.68
	Persistent subclinical hypocalcemia	0.21 ± 0.05	0.09 ± 0.04	0.40	0.14–1.12	0.08
	Subclinical mastitis	0.24 ± 0.05	0.17 ± 0.04	0.69	0.31–1.56	0.37
	Positive CMT	0.10 ± 0.03	0.14 ± 0.04	1.45	0.52–4.08	0.48
	First AI status	0.30 ± 0.06	0.29 ± 0.06	0.94	0.44–2.01	0.86
	Other diseases	0.11 ± 0.04	0.09 ± 0.03	0.81	0.28–2.39	0.70
Total	Retained placenta	0.11 ± 0.03	0.06 ± 0.02	0.55	0.22–1.38	0.20
	Metritis	0.32 ± 0.04	0.17 ± 0.03	0.42	0.23–0.78	0.01
	Endometritis	0.17 ± 0.04	0.17 ± 0.04	1.03	0.52–2.04	0.93
	Ketosis D5	0.31 ± 0.04	0.26 ± 0.04	0.80	0.46–1.41	0.44
	Ketosis D10	0.31 ± 0.04	0.25 ± 0.04	0.75	0.42–1.71	0.31
	Subclinical hypocalcemia D0	0.38 ± 0.05	0.29 ± 0.04	0.65	0.37–1.15	0.14
	Subclinical hypocalcemia D4	0.29 ± 0.04	0.22 ± 0.04	0.70	0.38–1.30	0.26
	Persistent subclinical hypocalcemia	0.15 ± 0.03	0.06 ± 0.02	0.34	0.13–0.90	0.03
	Subclinical mastitis	0.18 ± 0.04	0.14 ± 0.03	0.73	0.37–1.44	0.36
	Positive CMT	0.09 ± 0.03	0.17 ± 0.03	2.06	0.95–4.47	0.07
	First AI status	0.44 ± 0.05	0.40 ± 0.05	0.88	0.51–1.50	0.63
	Other diseases	0.08 ± 0.02	0.07 ± 0.02	0.92	0.35–2.40	0.86

Abbreviations: NIMS = Non-supplemented control group; IMS = Injectable mineral supplementation group; Mean = Mean between groups; SE = Standard error; D0 = Calving day; D4 = Fourth day postpartum; D5 = Fifth day postpartum; D10 = Tenth day postpartum; CMT = California Mastitis Test.

**Table 3:** Effect of injectable mineral supplementation on somatic cell count (SCC) in Holstein dairy cows across first 90 days in milk.

Parity	Sample Size	Classification According to DIM Interval ^1^	Group (Mean ± SE)		*p*-Value
			NIMS	IMS	
Primiparous	53	1 to 20 DIM (×10^3^ cells/mL)	93 ± 26.2	141 ± 37.4	0.27
	62	21 to 40 DIM (×10^3^ cells/mL)	59 ± 10.2	68 ± 13.0	0.59
	67	41 to 60 DIM (×10^3^ cells/mL)	23 ± 3.2	45 ± 5.7	0.00
	58	61 to 80 DIM (×10^3^ cells/mL)	32 ± 5.5	47 ± 7.8	0.11
	72	81 to 100 DIM (×10^3^ cells/mL)	43 ± 7.5	40 ± 6.5	0.78
	53	101 to 120 DIM (×10^3^ cells/mL)	38 ± 6.2	45 ± 7.3	0.43
	14	121 to 140 DIM (×10^3^ cells/mL)	24 ± 12.5	34 ± 12.9	0.52
Multiparous	75	1 to 20 DIM (×10^3^ cells/mL)	70 ± 15.7	131 ± 31.7	0.06
	88	21 to 40 DIM (×10^3^ cells/mL)	49 ± 11.1	60 ± 12.6	0.51
	85	41 to 60 DIM (×10^3^ cells/mL)	40 ± 9.5	46 ± 10.2	0.61
	101	61 to 80 DIM (×10^3^ cells/mL)	51 ± 10.0	53 ± 10.8	0.86
	92	81 to 100 DIM (×10^3^ cells/mL)	45 ± 10.8	55 ± 12.0	0.49
	71	101 to 120 DIM (×10^3^ cells/mL)	42 ± 10.6	103 ± 27.5	0.02
	19	121 to 140 DIM (×10^3^ cells/mL)	76 ± 56.7	66 ± 35.6	0.95
Total ^2^	128	1 to 20 DIM (×10^3^ cells/mL)	77 ± 13.3	133 ± 23.5	0.03
	150	21 to 40 DIM (×10^3^ cells/mL)	53 ± 7.8	62 ± 9.1	0.43
	152	41 to 60 DIM (×10^3^ cells/mL)	31 ± 4.6	45 ± 6.1	0.05
	159	61 to 80 DIM (×10^3^ cells/mL)	43 ± 6.0	50 ± 7.1	0.41
	164	81 to 100 DIM (×10^3^ cells/mL)	43 ± 6.7	48 ± 6.7	0.64
	124	101 to 120 DIM (×10^3^ cells/mL)	40 ± 6.5	70 ± 11.8	0.02
	33	121 to 140 DIM (×10^3^ cells/mL)	43 ± 20.0	47 ± 16.2	0.82

Abbreviations: NIMS = Non-supplemented control group; IMS = Injectable mineral supplementation group; SCC = Somatic Cell Count; Mean = Mean between groups; SE = Standard error; DIM = Days in milk. ^1^ Indicates the postpartum time point at which the milk sample was collected. SCC was reported according to DIM categories (1–20, 21–40, 41–60, 61–80, 81–100, 101–120, and 121–140 DIM). ^2^ Total animals per parity: Primiparous: 100 (NIMS = 47 and IMS = 53); Multiparous: 146 (NIMS, *n* = 72; IMS, *n* = 74); Total: 246 (NIMS, *n* = 119; IMS, *n* = 127).

**Table 4:** Mean values (±SE) of metabolic and biochemical biomarkers in primiparous cows from the NIMS and IMS groups, with *p*-values for the effects of group, sampling time, and their interaction (group × sampling).

Parity	Biomarkers	Group (Mean ± SE)		*p*-Value		
		NIMS	IMS	Group	Sampling	G × S ^1^
Primiparous	BHB	0.71 ± 0.03	0.72 ± 0.04	0.86	0.25	0.49
	NEFA	0.92 ± 0.04	0.92 ± 0.04	0.92	0.01	0.62
	TP	8.18 ± 0.22	8.24 ± 0.27	0.86	0.00	0.65
	ALB	2.61 ± 0.03	2.59 ± 0.04	0.71	0.62	0.85
	GLOB	5.57 ± 0.23	5.65 ± 0.28	0.81	<0.00	0.53
	AST	35.75 ± 1.01	37.08 ± 1.14	0.38	0.07	0.48
	TB	0.18 ± 0.01	0.19 ± 0.01	0.47	0.32	0.52
	DB	0.22 ± 0.01	0.23 ± 0.01	0.60	0.19	0.45
	TRIGL	17.93 ± 0.78	18.00 ± 0.92	0.95	0.02	0.68
	COL	92.74 ± 2.76	97.00 ± 3.08	0.32	0.12	0.10
	GLU	61.38 ± 0.92	62.99 ± 1.04	0.25	0.26	0.16
Multiparous	BHB	0.80 ± 0.05	0.72 ± 0.04	0.18	<0.00	0.57
	NEFA	0.95 ± 0.05	0.90 ± 0.05	0.44	<0.00	0.85
	TP	8.35 ± 0.25	8.43 ± 0.25	0.83	<0.00	0.95
	ALB	2.67 ± 0.03	2.61 ± 0.03	0.21	0.90	0.54
	GLOB	5.69 ± 0.25	5.82 ± 0.25	0.73	<0.00	0.93
	AST	35.85 ± 0.83	34.93 ± 0.74	0.41	0.00	0.65
	TB	0.21 ± 0.02	0.20 ± 0.02	0.70	0.01	0.92
	DB	0.23 ± 0.01	0.23 ± 0.01	0.99	0.04	0.91
	TRIGL	20.04 ± 0.86	18.14 ± 0.80	0.18	<0.00	0.20
	COL	94.43 ± 2.19	90.78 ± 1.95	0.21	0.06	0.78
	GLU	61.84 ± 0.99	63.91 ± 0.91	0.13	0.00	0.06
Total	BHB	0.76 ± 0.03	0.72 ± 0.03	0.33	<0.00	0.29
	NEFA	0.93 ± 0.03	0.91 ± 0.03	0.63	<0.00	0.84
	TP	8.23 ± 0.18	8.35 ± 0.19	0.65	<0.00	0.75
	ALB	2.64 ± 0.02	2.60 ± 0.02	0.22	0.60	0.68
	GLOB	5.61 ± 0.19	5.75 ± 0.20	0.60	<0.00	0.74
	AST	35.78 ± 0.66	35.63 ± 0.65	0.88	<0.00	0.56
	TB	0.19 ± 0.01	0.20 ± 0.01	0.96	0.02	0.91
	DB	0.23 ± 0.01	0.23 ± 0.01	0.62	0.04	0.92
	TRIGL	19.07 ± 0.60	18.09 ± 0.60	0.25	<0.00	0.69
	COL	93.70 ± 1.73	93.04 ± 1.69	0.79	0.01	0.91
	GLU	61.68 ± 0.62	63.63 ± 0.61	0.03	0.02	0.05

Abbreviations: NIMS = Non-supplemented control group; IMS = Injectable mineral supplementation group; Mean = Mean between groups; SE = Standard error; ALB = albumin; AST = aspartate aminotransferase; DB = direct bilirubin; BHB = β-hydroxybutyrate; TB = total bilirubin; COL = cholesterol; GLU = glucose; GLOB = globulins; HPT = haptoglobin; NEFA = non-esterified fatty acid; TP = total protein; TRIGL = triglyceride. ^1^ Group × Sampling.

**Table 5:** Mean values (±SE) of oxidative stress and immune biomarkers in primiparous and multiparous cows from the NIMS and IMS groups according to sampling time (weeks relative to calving), with *p*-values for the effects of group, sampling time, and their interaction (group × sampling time).

Parity	Variables	Group (Mean ± SE)		*p*-Value		
		NIMS	IMS	Group	Sampling	G × S ^1^
Primiparous	HPT	5.77 ± 0.52	4.57 ± 0.60	0.14	0.61	0.92
	IgG	26.9 ± 1.71	33.4 ± 1.95	0.02	<0.00	0.47
	GPX	1141 ± 51.9	1161 ± 61.4	0.80	0.10	0.18
	TBARS	2.62 ± 0.30	1.95 ± 0.34	0.16	0.01	0.71
	GSH	3.44 ± 0.49	3.80 ± 0.66	0.76	0.07	0.85
	TAS	1.00 ± 0.01	0.98 ± 0.02	0.16	<0.00	0.42
Multiparous	HPT	4.81 ± 0.39	4.88 ± 0.34	0.90	0.15	0.20
	IgG	28.4 ± 2.41	37.2 ± 2.14	0.01	<0.00	0.27
	GPX	822 ± 46.5	961 ± 42.3	0.03	0.05	0.78
	TBARS	2.03 ± 0.31	1.79 ± 0.27	0.58	<0.00	0.58
	GSH	3.49 ± 0.29	3.75 ± 0.29	0.54	0.29	0.36
	TAS	0.99 ± 0.02	0.97 ± 0.01	0.33	<0.00	0.14
Total	HPT	5.25 ± 0.28	4.81 ± 0.28	0.26	0.49	0.95
	IgG	27.7 ± 1.64	36.2 ± 1.60	0.00	<0.00	0.17
	GPX	963 ± 41.5	1028 ± 41.4	0.27	0.01	0.30
	TBARS	2.29 ± 0.22	1.87 ± 0.21	0.17	<0.00	0.82
	GSH	3.45 ± 0.23	3.75 ± 0.26	0.38	0.01	0.67
	TAS	1.00 ± 0.01	0.97 ± 0.01	0.11	<0.00	0.51

Abbreviations: NIMS = Non-supplemented control group; IMS = Injectable mineral supplementation group; Mean = mean between groups; SE = Standard error; HPT = Haptoglobin; IgG = Immunoglobulin G; GPX = Glutathione peroxidase; TBARS = Thiobarbituric acid-reactive substance; GSH = Reduced glutathione; TAS = Total antioxidant status. ^1^ Group × Sampling.

## Data Availability

The original data, including the dataset and Supplementary Materials presented in this study, are openly available in Zenodo at https://doi.org/10.5281/zenodo.18702107.

## Supplementary Materials

The following supporting information can be downloaded at: https://www.mdpi.com/article/10.3390/ani16060956/s1, Table S1: Ingredients, nutrients, and minerals (% of dry matter—DM) of diets offered at prepartum and postpartum during the experimental period in Holstein cows; Table S2: Mineral balance of prepartum diet for Holstein cows during the experimental period; Table S3: Mineral balance of postpartum diet for Holstein cows during the experimental period; Table S4: Metabolism analyses carried out in the group of 60 animals in the 7 moments of the transition period (3 weeks prepartum to 3 weeks postpartum).

## Abbreviations

The following abbreviations are used in this manuscript:

IMS	Injectable mineral supplementation
NIMS	Non-supplemented control group
DIM	Days in milk
CMT	California Mastitis Test
SCC	Somatic cell count
NEFA	Non-esterified fatty acid
BHB	Beta-hydroxybutyric acid
AST	Aspartate aminotransferase
Hp	Haptoglobin
IgG	Immunoglobulin G
TAS	total antioxidant status
TBARS	Thiobarbituric acid reactive substance
RBC	Red blood cell
GPx	Glutathione peroxidase
GSH	Glutathione
GLU	Glucose
Hb	Hemoglobin
