# Supplementary material for: Injectable Mineral Supplementation During the Transition Period Reduces Uterine Disease and Hypocalcemia and Enhances Humoral Immunity in Holstein Dairy Cows
Source: Animals (Basel). 2026 Mar 19;16(6):956. doi: 10.3390/ani16060956 (PMC13023310; doi:10.3390/ani16060956)
Supplement: Supplementary file 1 [file animals-16-00956-s001.zip › animals-4190426-supplementary.pdf]

Effects of injectable mineral supplementation on health, metabolic stress, and performance in Holstein cows during the transition period

Raquel S. Marques, Filipe A. Pinheiro, Clara S. Mori, Susan Suárez-Retamozo, Marcos Busanello, Rodrigo Almeida, Bruno S. Lima, Luc Durel and Viviani Gomes

Supplementary Table S1. Ingredients, nutrients, and minerals (% of dry matter – DM) of diets offered at prepartum and postpartum during the experimental period in Holstein cows.

Ingredients	Prepartum diet (%DM)	Postpartum diet (%DM)
Corn silage	67.84	42.75
Pre-dried	5.78	2.97
Cottonseed	8.87	5.26
Núcleo Pré-Parto AN 330 agMilk	3.18	-
Pós Parto SJ premix	-	0.43
Lysine	-	-
Methionine	0.09	-
Soybean meal	8.65	8.38
DDG	5.60	7.13
Corn	-	16.35
Citrus pulp	-	3.38
Pre-dried alfalfa silage	-	3.14
Canola meal	-	6.86
Enerfat (Kemin)	-	0.45
Smartamine	-	0.05
Magnesium oxide	-	0.31
Sodium bicarbonate	-	1.05
Salt	-	0.33
Calcitic limestone	-	1.17
**Total**	**100**	**100**
**Nutrients**		
Crude protein	14.77	17.4
Ethereal extract	4.26	4.40
Mineral residue	5.93	7.26
Raw fiber	-	-
ADF	25.70	17.86
Lignin	5.65	3.71
NDF	44.27	35.49
**Minerals**		
Calcium	0.41	1.11
Phosphor	0.38	0.47
Potassium	0.72	0.67

Source: São Jorge farms nutritionist, 2021
Abbreviations: DM = Dry matter; DDG = Dried Distillers Grains ; ADF = Acid-Detergent Fiber; NDF = Neutral Detergent Fiber

Supplementary Table S2. Mineral balance of prepartum diet for Holstein cows during the experimental period.

	Requirements (g/dL)						
	**Maintenance**	**Gestation**	**Lactation**	**Growth**	**Total required**	**Total provided**	**Balance**
**Macro minerals**							
Ca	9.8	7.8	0	1.4	18.9	24.7	5.8
P	11.2	4.4	0	0.8	16.4	27.8	11.5
Mg	3.6	0.3	0	0.1	4	8.4	4.5
Cl	12	0.9	0	0.1	13	81.8	68.8
K	70.9	0.9	0	0.3	72.1	85.2	13.1
Na	15.7	1.2	0	0.2	17.1	10.4	-6.7
S	21.7	0	0	0	21.7	48	26.3
**Trace minerals**							
Co	2.2	0	0	0	2.2	6.9	4.8
Cu	8.2	1.4	0	0.3	9.9	10	0.1
Fe	0	15.6	0	4.5	20.1	130.3	110.2
I	6.5	0	0	0	6.5	7	0.5
Mn	1.5	0.3	0	0.3	2	3.8	1.8
Se	3.3	0	0	0	3.3	6.6	3.4
Zn	54.2	10.6	0	3.2	68	207.4	139.3
Cr	6	0	0	0	6	0	0

Source: Animal Nutrition Laboratory, Department of Animal Science, Federal University of Paraná, 2021.
Abbreviations: Ca = Calcium; P = Phosphorus; Mg = Magnesium; Cl = Chlorine, K = Potassium; Na = Sodium; S = Sulfur; Co = Cobalt; Cu = Copper; Fe = Iron; I = Iodine; Mn = Manganese; Se = Selenium; Zn = Zinc; Cr = Chromium.

Supplementary Table S3. Mineral balance of postpartum diet for Holstein cows during the experimental period.

	Requirements (g/dL)						
	**Maintenance**	**Gestation**	**Lactation**	**Growth**	**Total required**	**Total provided**	**Balance**
**Macro minerals**							
Ca	19.6	0	36	0	55.6	121.2	65.6
P	22.1	0	31.1	0	53.2	64.7	11.5
Mg	7	0	3.8	-0.1	10.7	34.7	24
Cl	24.1	0	35	0	59.1	74.5	15.4
K	182.3	0	52.5	-0.6	234.2	174.7	-59.5
Na	31.5	0	14	-0.4	45.1	82.7	37.5
S	43.5	0	0	0	43.5	59.1	15.6
**Trace minerals**							
Co	43	0	0	0	4.3	8.9	4.6
Cu	9.3	0	1.4	0	10.7	16.9	6.2
Fe	0	0	35	-8.8	26.2	367.4	341.2
I	6.5	0	3.5	0	10	15.3	5.3
Mn	1.7	0	1	-0.5	2.2	7.3	5.1
Se	6.5	0	0	0	6.5	10.7	4.2
Zn	108.6	0	140	-6.2	242.5	312.7	70.3
Cr	0	0	0	0	0	0	0

Source: Animal Nutrition Laboratory, Department of Animal Science, Federal University of Paraná, 2021.
Abbreviations: Ca = Calcium; P = Phosphorus; Mg = Magnesium; Cl = Chlorine, K = Potassium; Na = Sodium; S = Sulfur; Co = Cobalt; Cu = Copper; Fe = Iron; I = Iodine; Mn = Manganese; Se = Selenium; Zn = Zinc; Cr = Chromium.

Supplementary Table S4. Metabolism analyses carried out in the group of 60 animals in the 7 moments of the transition period (3 weeks prepartum to 3 weeks postpartum). The table shows the commercial kits with their references and brands, used in the analysis of metabolism indicators of Holstein cows during the transition period.

Analysis	Brand	Reference	Interval of reference	Authors
Total bilirubin	Kovalent	1090075K	-	-
Direct bilirubin	Kovalent	1080075M	-	-
Cholesterol	Labtest	76-2	800 to 1 200 mg/L	Kaneko et al. [21]
Glucose	Labtest	133-1	450 to 750 mg/L	Kaneko et al. [21]
Triglycerides	Labtest	87-2/100	0 to 140 mg/L	Kaneko et al. [21]
Total protein	Labtest	99-250	68 000 to 86 000 mg/L	Smith et al. [22]
NEFA	Randox	FA115	Prepartum>0.3 mmol/L<br>Postpartum>0.6 mmol/L	Moyes et al. [23];<br>Ospina et al. [24]
BHB	Randox	RB1007	< 1.2 mmol/L	
Albumin	Biosystem	11573	30 300 to 35 500 mg/L	Kaneko et al. [21]
Calcium	Biosystem	11570	-	-
AST	Biosystem	11531	78 to 132 U/L	Kaneko et al. [21]

Abbreviations: NEFA = Non-Esterified Fatty Acids; BHB = Beta-Hydroxybutyric Acid; AST: Aspartate aminotransferase
